# Editorial: Synthetic biology approaches for biocatalytic production of value-added chemicals

**DOI:** 10.3389/fbioe.2025.1755163

**Published:** 2026-01-02

**Authors:** Huadong Peng, Sanjay K. S. Patel, Jung-Kul Lee

**Affiliations:** 1 Australian Institute for Bioengineering and Nanotechnology, The University of Queensland, Brisbane, QLD, Australia; 2 ARC Centre of Excellence in Synthetic Biology, The University of Queensland, St. Lucia, QLD, Australia; 3 Food and Beverage Accelerator (FaBA), The University of Queensland, St. Lucia, QLD, Australia; 4 Department of Biotechnology, Hemwati Nandan Bahuguna Garhwal University, Srinagar, India; 5 Department of Chemical Engineering, Konkuk University, Seoul, Republic of Korea

**Keywords:** biocatalysis, biochemicals, bioproduction, metabolic engineering, synthetic biology

The transition from petrochemical routes to biology-enabled manufacturing is now being driven as much by sustainability and circularity as by product novelty. This Research Topic, *Synthetic Biology Approaches for Biocatalytic Production of Value-Added Chemicals*, set out to capture that shift: not only “can we make X biologically?” but “can we design hosts, pathways, and cofactor systems so that making X becomes scalable, modular, and ultimately applicable to harder substrates and waste streams?” The eight contributions gathered here span exactly that arc - from pathway-centric metabolic engineering in bacteria and yeasts, to enabling toolkits for non-model hosts, to cofactor-recycling strategies, and finally to the emerging frontier of polymer upcycling using engineered biocatalytic systems. Together, they show that synthetic biology is maturing from single-pathway demonstrations to platform thinking.

We begin with the idea of aromatic amino acids as hubs. Shen et al. outline how tyrosine can be upgraded to an impressively broad portfolio, including resveratrol, L-DOPA, p-coumaric acid, caffeic acid, tyrosol, and more. They do so by walking through the chassis, precursor supply (PEP/E4P), and shikimate-pathway constraints, then concluding with the current industrial bottlenecks. That review is important because it frames tyrosine not just as a product but as a branch point that synthetic biologists can keep feeding with carbon and tailoring with enzymes (Shen et al.).

Moving from “what to make” to “where to make it,” Lee et al. deliver exactly what many in the field have been asking for: a modular, test-characterise-use genetic system for *W. ciferrii,* an underused but industrially promising yeast known for tetraacetyl phytosphingosine (TAPS) production. By benchmarking selectable markers, replication origins, promoters, and fluorescent reporters, and then demonstrating utility by boosting TAPS via ACC1 overexpression, they effectively elevate *W. ciferrii* to the status of an engineerable chassis, rather than a one-product curiosity. This is a classic synthetic-biology enabling paper and a key piece for anyone who wants to diversify eukaryotic hosts for lipidic or sphingolipid products (Lee et al.).

Synthetic biology, however, is not only about chassis; it is also about *biocatalytic elegance*. Singh et al. survey enzymatic routes to nicotinic acid and make a strong case for replacing harsh, low-selectivity chemical routes with nitrilases, engineered microbial pathways, and omics-guided enzyme discovery. What is notable here is their emphasis on pairing enzyme improvement (stability, catalytic efficiency) with smarter screening strategies such as metagenomics - a pattern we also see in other biocatalytic value-chain efforts. The take-home message is that vitamins and small nutraceuticals are every bit as amenable to synthetic-biology upgrading as are speciality chemicals, provided the right biocatalysts are identified and evolved (Singh et al.).

On the strictly metabolic-engineering side, Rehman et al. tackle a problem many have encountered: transferring a “standard” lycopene pathway into *B. subtilis* does not automatically work. By diagnosing a key enzymatic incompatibility (crtE) and swapping in a more suitable GGPPS from *Archaeoglobus fulgidus*, they then strengthen precursor supply via MEP-pathway engineering and medium optimisation, thereby increasing lycopene titres to the highest levels yet reported in *B. subtilis*. This paper is valuable beyond carotenoids - it reminds us that cross-kingdom pathways often fail due to a *single bad enzyme*, and that systematic swapping, combined with precursor boosting, remains one of the most reliable synthetic-biology recipes (Rehman et al.).

Efficient biocatalysis also relies on cofactors. Zhou et al. review the use of NAD(P)H oxidases to regenerate oxidised cofactors, thereby making dehydrogenase-based routes to rare sugars and fine chemicals economically feasible. By comparing H_2_O-forming and H_2_O_2_-forming NOXs and discussing protein-engineering levers (surface, active-site, and substrate-binding mutations), they position cofactor-recycling enzymes as first-class engineering targets, not afterthoughts, in value-added chemical production. Editorially, this is an important bridge between pathway design and process economics (Zhou et al.).

Not every contribution is about making the pathway; some are about making the *protein* that enables the assay or downstream application. Chen et al. show that the choice of molecular chaperone in *Escherichia coli* expression of an ABA-specific scFv changes not only soluble yield but also binding behaviour - sensitivity vs. specificity - through effects on folding and secondary structure. For synthetic biologists who rely on recombinant binders (for sensing, selection, or product analytics), this is a valuable reminder that host-level engineering can tune biorecognition tools just as much as sequence-level design (Chen et al.).

Two papers push the Research Topic toward harder feedstocks and circularity. Kim et al. present an ambitious CRISPR-Cas9 rewiring of *Yarrowia lipolytica* to block over-oxidation routes, then overexpress P450 alkane monooxygenases to convert inexpensive n-dodecane into medium-chain α,ω-diols - high-value monomers for polymers. It is a neat example of taking an oleaginous yeast that already handles hydrophobic substrates and converting it into a precise oxidiser by subtracting unwanted reactions and adding a strong entry enzyme. This is synthetic biology as substrate-range expansion (Kim et al.).

Finally, Abid et al. review biorecycling of polyethylene (PE), arguably one of the most intractable waste streams. What fits our Research Topic especially well is their framing: PE must first be made legible to biology (pretreatment, oxidation, depolymerisation), after which engineered microbes and metabolic-engineering strategies can upcycle the resulting fragments into PHAs, wax esters, diacids, or other value-added products. Seen alongside the *Y. lipolytica* alkane work, this review highlights a future where synthetic-biology toolkits for hydrophobic and recalcitrant substrates become central to circular bioeconomy strategies (Abid et al.).

Taken together, these articles do more than showcase individual advances. A few cross-cutting themes emerge:

1. From model to non-model hosts. Several studies deliberately equip yeasts beyond *S. cerevisiae* (*W. ciferrii* and *Y. lipolytica*) (Kim et al.; Lee et al.) with the genetic plumbing needed for iterative engineering, which will be essential for products that demand eukaryotic metabolism or hydrophobic-substrate handling.
2. Tightening the biocatalytic economy. Cofactor regeneration and single-enzyme bottleneck fixing are presented not as side notes but as main levers to make pathways viable at scale (Rehman et al.; Zhou et al.).
3. Toward circular and difficult carbon. Alkane-to-diol conversion and PE biorecycling show that synthetic biology is now being pointed at substrates previously left to thermochemical processing (Abid et al.; Kim et al.).
4. Enabling analytics and detection. Better expression of functional scFvs improves the whole pipeline because it supports high-throughput screening and quality control of engineered pathways (Chen et al.).

We hope this Editorial helps readers see the Research Topic as a coherent snapshot of where the field is going: leveraging synthetic-biology principles - modular parts, engineerable chassis, cofactor-aware pathway design, and application to unconventional feedstocks - to deliver value-added chemicals in a way that is greener and more versatile than incumbent methods. As Research Topic Editors, we also encourage future submissions that connect these strands at the process level (continuous fermentation, *in situ* product removal, waste-to-product chains), so that the impressive molecular and strain-level innovations reported here can move even faster toward deployment.

